# O-GlcNAcylation Links ChREBP and FXR to Glucose-Sensing

**DOI:** 10.3389/fendo.2014.00230

**Published:** 2015-01-13

**Authors:** Fadila Benhamed, Gaelle Filhoulaud, Sandrine Caron, Philippe Lefebvre, Bart Staels, Catherine Postic

**Affiliations:** ^1^U1016, Institut Cochin, INSERM, Paris, France; ^2^UMR 8104, CNRS, Paris, France; ^3^Sorbonne Paris Cité, Université Paris Descartes, Paris, France; ^4^European Genomic Institute for Diabetes (EGID), Lille, France; ^5^UMR 1011, INSERM, Lille, France; ^6^Univ Lille 2, Lille, France; ^7^Institut Pasteur de Lille, Lille, France

**Keywords:** ChREBP, FXR, glucose-sensing, O-GlcNAcylation, liver metabolism

## Abstract

Accumulating evidence suggests that O-GlcNAc transferase, an enzyme responsible for O-GlcNAc post-translational modification acts as a nutrient sensor that links glucose and the hexosamine biosynthetic pathway to the regulation of transcriptional factors involved in energy homeostasis. In liver, glucose signaling is mediated by carbohydrate response element-binding protein (ChREBP), which stimulates glycolytic and lipogenic gene expression through its binding on a specific ChoRE DNA sequence. Modulation of ChREBP by O-GlcNAcylation increases its DNA binding affinity and its activity. ChREBP transcriptional activity also depends on the presence of several other co-factors and transcriptional factors. Among them, the nuclear Farnesoid X Receptor (FXR), a key transcription factor of bile acid metabolism involved in the gut–liver axis homeostasis was recently shown to directly interact with ChREBP, acting as a repressor on the ChoRE of glycolytic genes. Interestingly, similarly to ChREBP, FXR is O-GlcNAcylated in response to glucose. This review discusses the importance of ChREBP and FXR modifications through O-GlcNAcylation in liver and how glucose can modify their mutual affinity and transcriptional activity.

## Introduction

The liver plays a central role in the control of energy homeostasis. In the liver, glucose does not only serve as an energy source but also acts as a signaling molecule to control the expression of key genes of glucose, fatty acid, and bile acid metabolism. Once in the hepatocyte, glucose is converted into glucose 6-phosphate (G6P) by the glucokinase enzyme (GK) leading, in turn, to the activation of glycolytic and lipogenic enzymes including l-pyruvate kinase (l-PK), acetyl-CoA carboxylase (ACC), and fatty-acid synthase (FAS). The positive effects of glucose on gene expression are mediated by the transcription factor carbohydrate-responsive element-binding protein (ChREBP). ChREBP, which belongs to the Mondo family of bHLH/Zip transcription factors, is a large protein (864 a.a) that contains several regulatory domains including a nuclear localization signal (NLS, amino acids 158–173) near the N-terminus, polyproline domains, a bHLH/LZ domain (amino acids 660–737), and a leucine zipper-like (Zip-like) domain (amino acids 807–847) (1). A conserved consensus sequence, named carbohydrate response element (ChoRE), the ChREBP-binding site, is required for glucose responsiveness. Modulation of ChREBP expression and/or activity by glucose occurs at multiple levels. In the presence of high glucose concentrations, ChREBP mRNA levels are increased (2, 3). ChREBP is also regulated at the post-translational level: in response to high glucose concentrations, ChREBP translocates into the nucleus (4) where the protein undergoes several post-translational modifications (PTMs), including acetylation and O-GlcNAcylation, which stimulates ChREBP activity and affinity for ChoRE sequences (5–7). The O-GlcNAc modification requires the hexosamine biosynthetic pathway (HBP): in response to high glucose concentrations, HBP synthesizes *N*-acetyl-glucosamine (UDP-GlcNAc), an obligatory substrate for β-*N*-acetylglucosaminyltransferase (OGT), a key enzyme allowing O-GlcNAcylation of proteins (8). This modification is reversible and is able to alter several protein properties such as stability, degradation, and/or modulation of transcriptional activity. Recently, key transcription factors involved in energy homeostasis, including ChREBP, have been reported to be modified by O-GlcNAc in liver. Among them, the nuclear receptor Farnesoid X receptor (FXR) (9) is a regulator of gene expression involved in bile acid synthesis and transport in the liver and the intestine (10). Interestingly, FXR was also recently reported to be involved in the control of glucose homeostasis via its direct interaction with ChREBP (9, 11). This review will discuss how ChREBP and FXR, both regulated by O-GlcNAcylation, modulate the signaling pathway that controls glucose homeostasis.

## ChREBP: A Key Regulator of Glucose Homeostasis

The discovery of ChREBP as key regulator of glycolysis and lipogenesis has shed light on the mechanism by which glucose transcriptionally regulates gene expression. ChREBP stimulates the expression of several genes involved in glucose and lipid metabolism such as l-PK, FAS, and ACC not only in the liver (3, 12) and also in adipose tissue (13) and in pancreatic β cells (14). ChREBP directly binds a conserved consensus sequence, ChoRE, present on the promoter region of its target genes (15, 16). The ChoRE sequence is composed of a tandem E box element separated by 5 nucleotides (5’-CACGTGnnnnnCACGTG-3’). ChREBP interacts with Max-like protein X (Mlx), its functional partner to form a heterodimer. The association of two heterodimers is necessary to bind the ChoRE motif and to provide a transcriptional complex regulated by glucose (17).

### Several key glucose metabolites activate ChREBP in response to glucose

The regulation of ChREBP activity by glucose is complex and brings in different steps [see Ref. (18) for review]. The laboratory of K. Uyeda was the first to describe a mechanism of activation dependent on a glucose metabolite. Kabashima et al. (19) demonstrated that xylulose-5-phosphate (X5P), a metabolite of the pentose phosphate pathway (PPP), is central for ChREBP translocation and DNA binding activity in response to glucose. Under high glucose concentrations, X5P activates the protein phosphatase PP2A, which dephosphorylates ChREBP on the serine residue 196 (Ser196), allowing its translocation to the nucleus. In a second step also occurring in a X5P and PP2A-dependent manner, ChREBP is dephosphorylated on the threonine residue 666 (Thr666) leading to its binding to DNA and to transactivation (19). However, this mechanism is controversial as several distinct hypotheses were proposed to explain the glucose-mediated activation of ChREBP [see Ref. (18) for review]. For instance, a structure–function analysis of the ChREBP protein identified an N-terminal domain, named the glucose-sensing module (GSM), a highly conserved sequence through evolution (20). The GSM contains two domains, the low glucose inhibitory domain (LID, residues 1–197) and the glucose-response activated conserved element, residues 197–298 (GRACE), both implicated in the regulation of ChREBP in response to glucose (21). Under low glucose concentrations, the GRACE domain is inhibited by the LID domain, leading to a lack of induction of ChREBP activity. Under high glucose concentrations, the inhibitory effect of the LID is relieved, thereby allowing the GRACE domain to stimulate ChREBP activity. In agreement with this hypothesis, deletion of the 196 first amino acids encompassing most of the LID yields a constitutive active form of ChREBP, independent of glucose concentrations (21). Interestingly, McFerrin and Atshley (22) identified a G6P binding pocket using structure prediction of the ChREBP protein. G6P, produced by the GK enzyme, after binding onto the GSM could induce a conformation change, dissociating the LID from the GRACE domain and therefore supporting ChREBP transactivation. More importantly, G6P could “open/derepress” the ChREBP protein structure allowing interaction with co-activators such as CBP and p300 (22). Arguments in favor of a role for G6P in activating ChREBP in hepatocytes and other cell types were reported. Overexpression of glucose-6-phosphate dehydrogenase (G6PDH), a rate limiting enzyme of the PPP in the pancreatic β cell line INS1 deprives cells from G6P and inhibits ChREBP transcriptional activity. In contrast, G6P accumulation driven by the specific inhibition of G6PDH activity increases ChREBP transcriptional activity in these cells (23). Using G6PDH overexpression and silencing approaches in hepatocytes, our laboratory showed that G6P, but not X5P, is required for ChREBP translocation to the nucleus and transactivation, suggesting that G6P is necessary and sufficient to induce ChREBP activity (24). The glucose-mediated activation of ChREBP remains complex and additional studies will be required to elucidate the exact contribution of the proposed metabolites. A step forward concerning ChREBP regulation was recently made when Herman and colleagues identified a novel variant of ChREBP named ChREBP-β (13). This variant arises from an alternative promoter located in exon1b of the ChREBP gene. This new transcript, which results from the splicing of exon 1b to exon 2, is translated at the next start-site located in exon 4 and produces a shorter protein of 687 amino acids (ChREBP-β) compared to the full-length protein ChREBP, re-named ChREBP-α (13). According to the hypothesis raised by Herman and co-workers, glucose metabolism (potentially G6P) would first induce the transcription of ChREBP-α. ChREBP-α would in turn bind the ChoRE identified in exon1b to enhance ChREBP-β transcription. In adipose tissue, ChREBP-β was described as a much more potent transcriptional regulator than ChREBP-α. In the liver, ChREBP-β expression seems to be less sensitive than that of ChREBP-α to nutritional regulations (fasting versus refeeding). However, the physiological contribution of ChREBP-β to the glucose-induced transcriptional response in the liver remains to be determined.

### A central role for ChREBP in regulating hepatic glycolysis and lipogenesis

Convincing *in vitro* and *in vivo* evidences revealed that ChREBP is required for the induction of glycolytic and lipogenic genes in response to glucose (3, 12). Stimulation of primary cultured hepatocytes with high glucose concentrations (25 mM) leads to the induction of ChREBP expression and activity allowing stimulation of its target genes (25). In contrast, inhibition of ChREBP expression by a siRNA approach prevents this induction and blunts the accumulation of lipids in response to glucose (3). Importantly, global inactivation or liver-specific inhibition of ChREBP leads to a decrease in glycolytic and lipogenic gene expression associated with a significant decrease in triglyceride synthesis under both physiological and pathophysiological conditions (12, 26). The mirror experiment in which the ChREBP protein was overexpressed through an adenoviral approach in liver of mice led to an exacerbation of the glycolysis and lipogenesis pathways associated with the development of hepatic steatosis (27).

## FXR: A New Modulator of Glucose Homeostasis

Once activated by its ligands such as natural bile acids, FXR binds, alone or with its partner Retinoid X receptor (RXR), onto its response elements (FXRE) to regulate its target genes. While largely implicated in the transcriptional control of genes controlling bile acid metabolism, FXR also recently emerged as a novel modulator of glucose homeostasis (28, 29). FXR is necessary for the control of blood glucose concentrations in response to starvation in mice. FXR knockout mice (FXR^−/−^, whole body inactivation) were reported to be hypoglycemic in response to a short time (6 h) fasting. This phenotype can be, in part, explained by an alteration of the expression of phosphoenolpyruvate carboxykinase (PEPCK), a key enzyme of gluconeogenesis. Interestingly, the response to longer fasting (24–48 h) was not affected in the absence of FXR, suggesting a defective adaptative response in FXR^−/−^ mice. Surprisingly, activation of FXR by GW4064, a specific synthetic ligand, did not increase PEPCK expression in primary mouse hepatocytes (30). However, primary human and rat hepatocytes stimulated with GW4064 displayed an increase in PEPCK expression that was correlated with enhanced glucose output (31) suggesting either species differences or dependence on changes such as nutritional or environmental stimuli.

Interestingly, FXR^−/−^ mice respond more rapidly to high carbohydrate feeding with an accelerated induction of glycolytic and lipogenic genes without, however, any difference in ChREBP mRNA levels (29). In addition, nuclear translocation of ChREBP protein was not affected by FXR activation. FXR was shown to directly interact with the ChREBP protein in different cell lines. *In vitro* GST pulldown experiments showed that FXR interacts with ChREBP, irrespective of its ligation to GW4064. Analysis of FXR deletion mutants revealed that FXR interacts with ChREBP via its N-terminal activation function-1 (AF-1) domain (amino acids 1–127) and via the N-terminal part of its ligand-binding domain (amino acids 215–300) (11).

**Figure 1:**
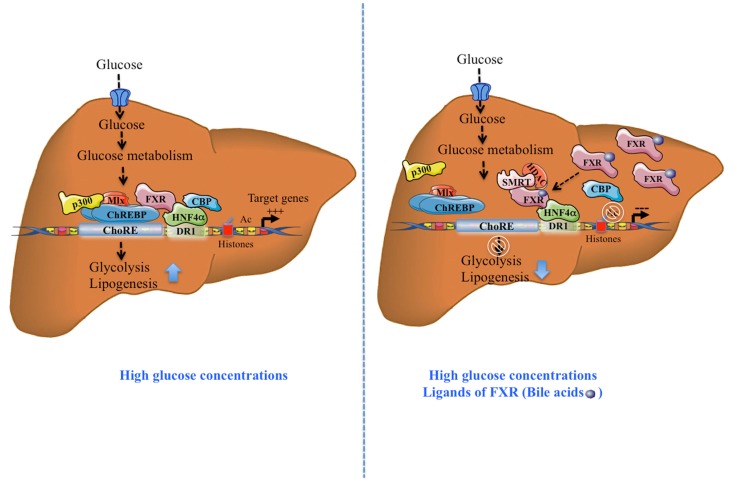
**Activation and transrepression of ChREBP-target genes by ChREBP and FXR**. After a meal, in the presence of high glucose concentrations, without FXR activation, ChREBP binds together with HNF4α, to ChoRE region of the l-PK promoter and transactivates gene expression, in part due to the recruitment of co-activators p300 and CBP. Due to its direct interaction with ChREBP and HNF4α, FXR interacts with this complex. The complex formation leads to the stimulation of the glycolytic and lipogenic pathways. The synergistic presence of high glucose concentrations and FXR ligands (bile acids, CDCA), activated FXR recruits the co-repressor SMRT. This recruitment leads to the release of ChREBP, CBP, and p300 leading to the inhibition of ChREBP-target gene expression. Tethered to the promoter through its interaction with HNF4α, FXR recruits the transcriptional co-inhibitor SMRT and represses transcription through the recruitment of HDACs and deacetylation of H3 histones. This effect leads to inhibition of the glycolytic and lipogenic pathways.

At the functional level, treatment of primary hepatocytes with the FXR agonist GW4064 decreased the glucose-induced expression of l-PK, ACC, and FAS. This inhibition was prevented in FXR^−/−^ hepatocytes. Importantly, using a ChoRE luciferase promoter construct, the authors reported that FXR transfection and/or activation prevented the stimulation of ChoRE-driven target genes. Gel shift analysis revealed that FXR was indeed able to bind to the L3 site (contained within the ChoRE) but not to the L4 site of l-PK promoter (29). These results were confirmed in the immortalized human hepatocyte (IHH) cell line: when activated by either its most potent natural ligand (CDCA: chenodeoxycholic acid) or synthetic agonists (GW4064; INT747; and WAY362450), FXR was able to bind the l-PK promoter. Finally, using chromatin immunoprecipitation (ChIP) assays, Caron et al. (11) were able to demonstrate the concomitant recruitment of ChREBP, HNF4α, p300, CBP, and FXR on the genomic L4/L3 region of the l-PK promoter in the presence of high glucose concentrations. According to this model, agonist-mediated activation of FXR leads to the release of CBP and p300, while allowing the recruitment of the co-repressor SMRT (Figure 1). This study reveals that FXR acts as a transrepressor and provides a novel mechanism by which FXR directly controls ChREBP-dependent genes, such as the l-PK gene.

The structural base of the repressive activity of FXR on ChREBP activity may rely on the existence of the LxQLLT motif, called the nuclear receptor box (NRB) within the ChREBP protein (22). This NRB matches the consensus LXXLL motif primarily found in co-activators of nuclear receptors that confers agonist-induced binding to nuclear receptors suggesting a potential ligand-dependent interaction between ChREBP and nuclear receptors such as FXR (22) and recruitment of FXR on ChoRE-bound ChREBP. SMRT tethering to the FXR–ChREBP complex could then occur through the second co-activator binding motif specifically found in FXR (32), although this awaits formal investigation. It would be of interest to mutate this NRB motif and study the modification of interaction between ChREBP and FXR as well as the consequences on the transcriptional regulation of the l-PK gene. One can also speculate that under appropriate conditions, ChREBP might serve as a FXR co-regulator, hence conferring glucose responsiveness to FXR. Such a possibility could be investigated by high resolutive genomic binding studies such as ChIP-Exo assays. Another hypothesis is that glucose metabolism could act as a signal that activates FXR independently of ChREBP via PTMs such as O-GlcNAcylation, as discussed below.

## ChREBP and FXR are O-GlcNAcylated

### O-GlcNAcylation stabilizes the ChREBP protein and stimulates its transcriptional activity

Approximately 2–5% of total glucose in the cell is used through the HBP. O-GlcNAcylation is a dynamic reaction catalyzed by two enzymes: (i) O-GlcNAc transferase (OGT), which adds a monosaccharide to serine/threonine residues of target proteins; (ii) the *O-GlcNAc hydrolase* (OGA), which hydrolyzes the monosaccharide. Sakiyama and co-workers (33) first showed that when Hepa1–6 hepatoma cells are treated with PUGNAC, a drug that increases O-GlcNAc, the transcriptional activity of ChREBP is exacerbated under high glucose concentrations, without any change in protein levels. In contrast, in cells treated with DON (6-diazo-5-oxo-l-norleucine), a drug that decreases O-GlcNAc, the stimulatory effect of high glucose on ChREBP activity is prevented (33). Our laboratory showed that ChREBP directly interacts with OGT in HEK293 cells and hepatocytes (6). ChREBP is O-GlcNAcylated in hepatocytes treated with high concentrations of glucose or glucosamine and in the liver of refed mice, demonstrating a nutritional regulation of ChREBP O-GlcNAcylation (ChREBP^OG^). In mouse hepatocytes, overexpression of OGA led to an inhibition of ChREBP-target genes associated with a decrease of lipid droplets under high glucose concentrations. *In vivo*, OGT overexpression in mouse liver was associated with an increase of ChREBP^OG^, correlated with the induction of l-PK expression and with ChREBP recruitment to the l-PK promoter. OGT overexpression also increased ChREBP protein content without modifying ChREBP mRNA levels suggesting that the protein may be stabilized by O-GlcNAcylation (6). Interestingly, in a follow-up study, Ido-Kitamura and co-workers suggested that ChREBP poly-ubiquitination (ChREBP^ub^) was reduced when ChREBP is O-GlcNAcylated (7) suggesting that these two PTMs may interfere to regulate ChREBP stability.

### FXR is regulated by O-GlcNAcylation

While the impact of nutrients and glucose on bile acid homeostasis is not fully understood (10), it was recently shown that FXR can be modified through O-GlcNAcylation in response to high glucose concentration level (9). *Berrabah* et al. revealed that FXR is modified by O-GlcNAcylation through its interaction with OGT, which catalyzed this reaction in response to high glucose level. O-GlcNAcylation leads to an increase of FXR protein stability, transcriptional activity, and chromatin binding through SMRT inactivation. O-GlcNAcylation of FXR occurs on serine 62 (Ser62) within the AF-1 domain. In agreement, mutation of Ser62 decreased FXR O-GlcNAcylation that correlated with an inhibition of its transcriptional activity. *In vivo*, nutritional experiments reveals that FXR is O-GlcNAcylated under fed conditions, which correlates with an induction of its target genes (Shp, Cyp7A1) and a decrease in hepatic bile acid content. Interestingly, a recent study reported that FXR can also be modified by SUMOylation (34). Ligand-dependent SUMOylation of FXR leads to a decrease of FXR transcriptional activity and consequently to a down regulation of its target genes. Interestingly, O-GlcNAcylation (Ser62) and sumoylation (Lys122) of FXR occur within the same domain, the A/B-domain known to play gene-specific role in transactivation and cofactor recruitment (11). It would be of interest to determine whether O-GlcNAcylation of FXR prevents and/or interferes with its SUMOylation and vice versa.

### Relevance of ChREBP and/or FXR O-GlcNAcylation to physiopathology

**Figure 2:**
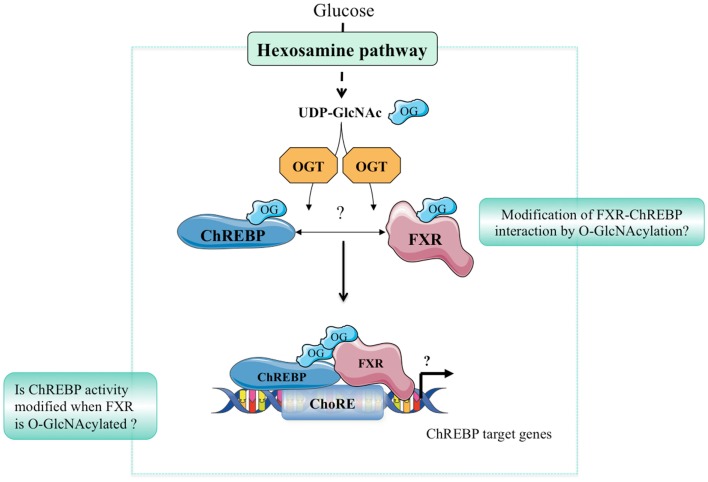
**Hypothetical model of ChREBP and FXR interaction**. In response to high glucose concentrations, the hexosamine biosynthesis (HBP) pathway is activated leading to UDP-GlcNAc production. ChREBP and FXR are in turn O-GlcNAcylated through direct binding with the OGT enzyme. O-GlcNAcylation of ChREBP and FXR may represent an important feature of their interaction. The physiological or pathophysiological consequences of such a modification remains, however, unknown.

Hyperglycemia and diabetes result in an increased flux through the HBP, which, in turn, increases PTM of Ser/Thr residues of proteins by O-GlcNAcylation. Altered O-GlcNAc signaling has been implicated in the pathogenesis of diabetes and may play an important role in its complications including non-alcoholic fatty liver disease (NAFLD), diabetic nephropathy, and/or retinopathy (35). Indeed, we have reported that the hepatic content of ChREBP^OG^ is increased in liver of diabetic db/db mice, and correlated to the pathophysiology of hepatic steatosis in this mouse model. OGA overexpression in the liver of db/db mice reduced ChREBP^OG^ concentrations leading to an inhibition of its target genes involved in *de novo* lipogenesis. Consequently, hepatic steatosis was prevented and correlated to an improvement of several physiological parameters (improved glucose tolerance and insulin sensitivity). The improved phenotype in OGA-treated db/db mice was also associated with a significant decrease in O-GlcNAcylation of the transcriptional co-activator CRTC2 (6) involved in the control of gluconeogenic genes (36). Recently, the role of ChREBP^OG^ in diabetic nephropathy was also investigated (37). Treatment with high glucose concentrations increased cellular O-GlcNAc and ChREBP^OG^ levels in mesangial cells compared with low glucose concentrations. PUGNAc treatment increased ChREBP-target expression in mesangial cells, whereas DON blunted the stimulatory effect of high glucose. Mechanistically, O-GlcNAc augmented protein stability, transcriptional activity, and nuclear translocation of ChREBP in these cells, leading to an exacerbated lipid accumulation. Importantly, in a pathophysiological context, ChREBP^OG^ was elevated in mesangial cells from streptozotocin-induced diabetic rats. Altogether, this study suggests that the hyperglycemia-mediated induction of ChREBP O-GlcNAcylation in mesangial cells may drive excess lipid accumulation and fibrosis, characteristic features of diabetic nephropathy (37). The potential contribution of FXR O-GlcNAcylation to the pathophysiology of liver and/or of other organs has not yet been addressed. Interestingly, FXR deficiency was previously reported to improve several of the metabolic abnormalities observed in ob/ob mice. Indeed, FXR^−/−^ mice crossed on ob/ob background are less obese, more tolerant to glucose and more sensitive to insulin than controls (38). The finding that FXR is modified by O-GlcNAcylation (9) further links bile acid metabolism to nutrient availability as observed in human in physiology (39), but also in a context of metabolic dysfunctions such as type 2 diabetes (40). In fasting–refeeding experiments, an FXR-dependent correlation between hepatic bile acid content, FXR transcriptional activity, and plasma glucose concentration has been established, suggesting that O-GlcNAcylation of FXR might regulate bile acid production (9). However, this awaits a formal demonstration using O-GlcNAcylation-deficient FXR *in vivo*. Importantly, the concomitant regulation of ChREBP and FXR by O-GlcNAcylation in liver cells in response to hyperglycemia may trigger and/or enhance their physical interaction, modulating in turn the transcriptional regulation of their common target genes involved in glycolysis, lipogenesis, and/or bile acid metabolism (Figure 2). Interestingly, FXR was reported to interact with the ChREBP protein through its AF-1 domain (11), a domain also shown to be the site of FXR O-GlcNAcylation (9). Further analysis of this interaction in response to high glucose concentrations, as well as the identification of O-GlcNAc residues within the ChREBP protein should provide a better understanding of the relevance of the coordinated O-GlcNAcylation of ChREBP and FXR under physiological and pathophysiological conditions (Figure 2).

## Conflict of Interest Statement

The authors declare that the research was conducted in the absence of any commercial or financial relationships that could be construed as a potential conflict of interest. The Associate Editor Tarik Issad declares that, despite being affiliated to the same institution as the authors Fadila Benhamed, Gaelle Filhoulaud and Catherine Postic, the review process was handled objectively and no conflict of interest exists.

## Abbreviations

ACC, acetyl-CoA carboxylase; AF-1, activation function-1; ChIP, chromatin immunoprecipitation; CBP, CREB-binding protein; ChoRE, carbohydrate response element; ChREBP, carbohydrate-responsive element-binding protein; FAS, fatty-acid synthase; FXR, farnesoid X receptor; FXRE, FXR response element; G6P, glucose-6-phosphate; GSM, glucose-sensing module; GK, glucokinase; G6PDH, glucose 6-phosphate dehydrogenase; GRACE, glucose-response activated conserved element; HBP, hexosamine biosynthetic pathway; LID, low glucose inhibitory domain; l-PK, l-pyruvate kinase; Mlx, max-like protein X; NAFLD, non-alcoholic fatty liver disease; NRB, nuclear receptor box; OGT, β-*N*-acetylglucosaminyltransferase; p300, histone acetyl transferase p300; PPP, pentose phosphate pathway; PEPCK, phosphoenolpyruvate carboxykinase; PTM, post-translational modification; UDP-GlcNAc, *N*-acetyl-glucosamine; NLS, nuclear localization signal; RXR, retinoid X receptor; X5P; xylulose-5-phosphate.
